# Examining the Impact of Alcohol Labels on Awareness and Knowledge of National Drinking Guidelines: A Real-World Study in Yukon, Canada

**DOI:** 10.15288/jsad.2020.81.262

**Published:** 2020-05-03

**Authors:** Nour Schoueri-Mychasiw, Ashini Weerasinghe, Kate Vallance, Tim Stockwell, Jinhui Zhao, David Hammond, Jonathan McGavock, Thomas K. Greenfield, Catherine Paradis, Erin Hobin

**Affiliations:** ^a^ Public Health Ontario, Toronto, Ontario, Canada; ^b^ Canadian Institute for Substance Use Research, University of Victoria, Victoria, British Columbia, Canada; ^c^ School of Public Health and Health Systems, University of Waterloo, Waterloo, Ontario, Canada; ^d^ Children’s Hospital Research Institute of Manitoba, University of Manitoba, Winnipeg, Manitoba, Canada; ^e^ Alcohol Research Group, Public Health Institute, Emeryville, California, United States; ^f^ Canadian Centre on Substance Use and Addiction, Ottawa, Ontario, Canada; ^g^ Dalla Lana School of Public Health, University of Toronto, Toronto, Ontario, Canada

## Abstract

**Objective:**

Alcohol labels are one strategy for communicating health information to consumers. This study tested the extent to which consumers recalled alcohol labels with national drinking guidelines and examined the impact of labels on awareness and knowledge of the guidelines.

**Method:**

A quasi-experimental study was conducted in two jurisdictions in northern Canada examining the impact of labels on the following outcomes: unprompted and prompted recall of the drinking guideline label message, awareness of the drinking guidelines, and knowledge of the daily and weekly recommended drink limits. The intervention site applied labels with national drinking guidelines, a cancer warning, and standard drink information to alcohol containers in its liquor store, whereas the comparison site did not apply these labels. In total, 2,049 cohort participants in both sites were recruited to complete surveys before and at two time points after the intervention. Changes in outcomes were examined using generalized estimating equations.

**Results:**

After the intervention, unprompted and prompted recall of the drinking guideline label message increased more in the intervention versus comparison site (adjusted odds ratio [AOR] = 10.8, 95% CI [0.9, 127.6]; AOR = 7.0, 95% CI [3.3, 14.9], respectively). Awareness of the drinking guidelines increased 2.9 times more in the intervention versus comparison site (AOR = 2.9, 95% CI [2.0, 4.3]). In addition, knowledge of the daily and weekly drink limits increased 1.5 and 1.4 times more in the intervention versus comparison site, respectively (daily: AOR = 1.5, 95% CI [1.0, 2.1]; weekly: AOR = 1.4, 95% CI [1.0, 2.0]).

**Conclusions:**

Enhanced alcohol labels get noticed and may be an effective population-level strategy for increasing awareness and knowledge of national drinking guidelines.

Globally, alcohol use is the seventh leading risk factor for disability and premature death (GBD 2016 Alcohol Collaborators, 2018) and is the leading risk factor among those ages 15–49 (World Health Organization [WHO], 2018a). Alcohol use is linked to more than 200 diseases, including at least seven types of cancer, and causes 3 million deaths per year globally (GBD 2016 Alcohol Collaborators, 2018; Rehm et al., 2017; WHO, 2018a). Recent estimates indicate that the prevalence of alcohol consumption and amounts consumed have increased globally, and will continue to rise (Manthey et al., 2019). Given the current trends, the total direct and indirect costs (e.g., healthcare, lost productivity, criminal justice) of alcohol use in developed countries, including Canada, exceed those from all illicit substances combined and are similar to or, by some estimates, greater than those for tobacco (Canadian Substance Use Costs and Harms Scientific Working Group, 2018). Population-level strategies to moderate alcohol use are therefore critical for improving public health.

National alcohol drinking guidelines exist in 37 countries to promote moderation and to reduce alcohol-related harms (Kalinowski & Humphreys, 2016). Drinking guidelines typically provide upper limits on the number of standard drinks that adults should not exceed in a day and/ or week. In Canada, the first nationally endorsed drinking guidelines were released in 2011, with the key guidelines recommending no more than 15 standard drinks in a week for men with no more than 3 on most days, and no more than 10 standard drinks in a week for women, with no more than 2 on most days (Butt et al., 2011). A “standard drink” in Canada is defined as 13.45 g or 17.05 ml of ethanol and is equivalent to the following: a 341 ml (12 oz.) can of 5% beer or cooler, a 142 ml (5 oz.) glass of 12% wine, and 43 ml (1.5 oz.) of 40% distilled alcohol (Butt et al., 2011). More than 22 million adults (78% of the population) in Canada drink alcohol (Statistics Canada, 2018), with 27% regularly exceeding the weekly limits and 39% exceeding the daily limits in 2008–2010, outlined in the guidelines, after adjusting for underreporting (Zhao et al., 2015). If Canadians who currently drink above the guidelines reduced their consumption to the recommended limits and all others maintained their current drinking patterns, overall consumption in Canada would be reduced by at least 50% (Stockwell et al., 2009).

To adhere to drinking guidelines, consumers must first be aware of and understand the recommended limits of alcohol intake. Public awareness and knowledge of drinking guidelines in Canada and internationally are low (Bowden et al., 2014; Buykx et al., 2018; De Visser & Birch, 2012; Livingston, 2012; McNally et al., 2019; Rosenberg et al., 2018). Mass media campaigns have failed to increase awareness and knowledge of these guidelines. For example, an evaluation of a Canadian social marketing campaign found that awareness of Canada’s low-risk drinking guidelines was approximately 19% at baseline (McNally et al., 2019), consistent with other estimates in Canada (Charbonneau et al., 2014; Fox, 2018). Awareness of the guidelines improved by 7% after the campaign, but no differences in knowledge of the recommended drink limits were observed (McNally et al., 2019). Strategies that extend beyond media advertising are needed to increase public awareness of national drinking guidelines. Increasing public awareness of national drinking guidelines is important, as recent data from a large population-based sample of Australian adults demonstrates a positive association between knowledge of recommended drink limits and a self-reported reduction in alcohol consumption, particularly among heavier drinkers (Islam et al., 2019).

Alcohol labels on product containers are one strategy for communicating health information to consumers at key points of contact—the point-of-purchase and -pour—and are recommended by national and international health organizations (Australia Department of Health, 2019; CCSA, 2007; UK Department of Health, 2007; WHO, 2017). Product labels are believed to influence behavior by gaining consumers’ attention, eliciting aversive reactions, and keeping the message in consumers’ minds (Brewer et al., 2019). Labels are appealing because of their relatively low cost to regulators, unparalleled reach among drinkers, and higher exposure among the heaviest drinkers (Greenfield, 1997). Laboratory and online experiments examining the optimal design of alcohol labels suggest that labels including a health warning, standard drink information, and national drinking guidelines could help consumers monitor their drinking and understand the extent to which this differs from the recommended guidelines (Blackwell et al., 2018; Hobin et al., 2018; Rosenberg et al., 2018).

Although Canada does not currently mandate health warning labels on alcohol containers, 47 other countries currently have implemented labels, with the majority requiring warnings cautioning about the risks of drinking while pregnant or while operating a vehicle (WHO, 2018b). Only eight countries mandate standard drink information on labels, and none mandate drinking guidelines (WHO, 2018b). The United Kingdom has a voluntary agreement with the alcohol industry for alcohol labels with pregnancy warnings, unit information, and drinking guidelines; however, recent studies found that these labels are poorly designed and include outdated drinking guideline information (Alcohol Health Alliance UK, 2017; Blackwell et al., 2018; Royal Society for Public Health, 2018). It remains unclear whether well-designed labels are an effective tool for communicating alcohol-related health risks, tracking alcohol consumption, and adhering to recommended drink limits.

This article is one of a series of articles (see Hobin et al., 2020; Vallance et al., 2020a; Weerasinghe et al., 2020) from a larger study aiming to test evidence-informed alcohol labels with a cancer warning, national drinking guidelines, and standard drink information. Using an experimental design, in a real-world setting, we tested whether alcohol labels are an effective population-level strategy for supporting more informed and safer alcohol use. The specific objectives of the current article are to (a) determine the extent to which consumers recalled alcohol labels with national drinking guidelines, (b) examine the impact of the alcohol labels on awareness and knowledge of national drinking guidelines, and (c) describe the level of support for alcohol labels with national drinking guidelines.

## Method

### Alcohol label intervention

**Figure 1.**
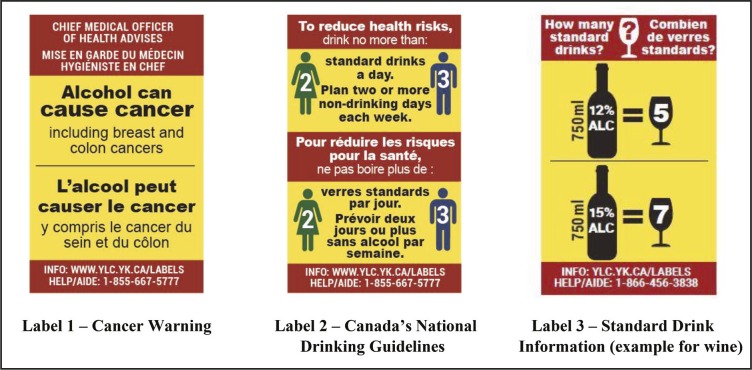
Intervention alcohol warning labels (actual size 5.0 cm × 3.2 cm each). The label intervention included three rotating labels: (a) a cancer warning, (b) national drinking guidelines, and (c) standard drink information (four separate labels were developed for wine, spirits, coolers, and beer; wine example shown above). *Note:* Alcohol containers sold in the liquor store in the intervention condition were each labeled with one of the three label options displayed above.

The alcohol label intervention included three rotating post-manufacturer labels with (a) a cancer warning, (b) national drinking guidelines, and (c) standard drink information (four separate labels were developed for wine, distilled spirits, coolers, and beer; Figure 1). Label development was informed by previous alcohol and tobacco labeling studies (Greenfield, 1997; Hammond, 2011; Hobin et al., 2018; Pettigrew et al., 2016; Strahan et al., 2002; Vallance et al., 2018) and by consultations with local and international health experts and community stakeholders. The labels were large (5.0 cm × 3.2 cm), used bright colors so they stood out on the containers, provided messages that are largely novel to consumers, and were rotated to avoid wear out (Hammond, 2011; Martin-Moreno et al., 2013). Moreover, the three label messages were designed to complement each other by providing a serious health message to grab consumer attention, and standard drink information and national drinking guidelines to support consumers in tracking consumption and adhering to recommendations. The labels were printed in Canada’s two official languages, English and French, and included a toll-free help line and a website linking to recommendations for minimizing alcohol-related risks. A social marketing campaign consisting of in-store signs, pamphlets, and radio spots was planned to run alongside the labels, as per effective labeling practices (Babor et al., 2010; Thomson et al., 2012; Vallance et al., 2018).

### Study design

A real-world quasi-experimental study was conducted among cohort participants recruited in liquor stores in the intervention site (Whitehorse, Yukon, Canada) and the comparison site (Yellowknife, Northwest Territories, Canada). These sites were selected because Yukon and Northwest Territories are the only two jurisdictions in Canada that require post-manufacturer alcohol labels to be applied to most alcohol containers sold in government-run retail liquor stores. Since 1991, labels in both jurisdictions warn consumers about drinking while pregnant, with an additional message in Northwest Territories cautioning against drinking and driving or operating machinery and that alcohol may cause health problems (Government of Northwest Territories, 2017; Government of Yukon, 2016). In addition, the one store in the intervention site and two stores in the comparison site are the only government-run liquor stores in both cities, and between them they account for approximately 50% of alcohol sales in these jurisdictions (Government of Northwest Territories, 2017; Government of Yukon, 2017). The intervention labels were scheduled to replace the original warning labels on all alcohol containers, except single-serve beer and cider (approximately 3% of sales), in the one liquor store in the intervention site for an 8-month period. The two liquor stores in the comparison site continued usual labeling practices (see Vallance et al., 2020a, for a detailed study protocol).

Two waves of surveys were scheduled in the intervention and comparison sites, 4 months before and 8 months after the intervention labels were implemented. The intervention labels with the cancer warning and national drinking guidelines were applied to alcohol containers in the intervention site starting November 20, 2017, with the standard drink label to be introduced shortly after. Liquor store staff were instructed to apply the intervention labels upright and to avoid covering manufacturer labels on the containers.

**Figure 2.**
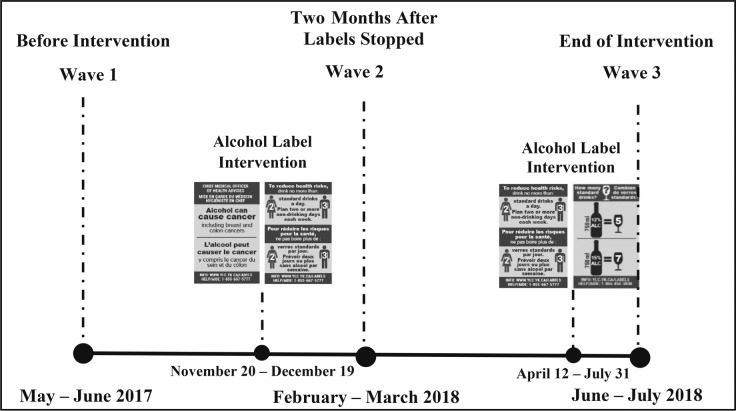
Modified study design due to interference from Canada’s alcohol industry

However, 1 month into the 8-month intervention period, the government in the intervention site paused its participation in the study because of pressure from Canada’s alcohol industry and stopped applying labels (Austen, 2018; Vallance et al., 2020a). Based on remaining label stock, approximately 47,000 cancer warning labels and 53,000 national drinking guidelines labels were applied to alcohol containers within the 1-month period. In April 2018, the government resumed its participation, with the caveat that the cancer label be excluded from the label rotation. Thus, the drinking guidelines labels were reinstated starting April 12, 2018, for an additional 3.5 months, and the standard drink labels were reinstated starting May 28, 2018, for 2 months. Approximately 117,000 drinking guidelines labels and 92,000 standard drink labels were applied to alcohol containers between April and July 2018. As a result of the interruption in the label intervention, the study design was modified (Figure 2). Wave 2 surveys were conducted starting mid-February 2018, 2 months after the government paused its participation, in order to capture the impact of the shortened intervention. Wave 3 surveys were conducted starting mid-June 2018 to the end of the intervention period in July 2018. The intended social marketing campaign was not implemented due to industry interference, with the exception of the project website and a media release at the time of the initial launch of the label intervention in November 2017.

### Recruitment and survey procedures

A prospective cohort of adult drinkers was recruited in Wave 1 by trained research assistants (RAs) as they exited the liquor stores in both sites, using a standard intercept technique of approaching every person that passed a pre-identified landmark in the liquor store. Participants were asked to complete a screener on a 10-inch tablet to identify eligibility status. Eligible participants provided consent and completed the Wave 1 survey on the tablet without RA assistance. In Waves 2 and 3, participants who provided their contact information were emailed survey instructions, a unique survey link, and an e-transfer as remuneration. In addition, due to attrition, the sample was replenished using Wave 1 recruitment protocols in both sites (see Vallance et al., 2020a, for the cohort structure). All survey periods lasted approximately 6 weeks, the surveys took approximately 18 minutes to complete, and survey measures were consistent across waves and sites. Study procedures were approved by the Research Ethics Boards at Public Health Ontario (ID 2017-010.04) and the University of Victoria (Protocol 17-161).

### Participants

Participants were adults of legal drinking age (≥19), residents of either the intervention or the comparison city, and, at the time of recruitment, were current drinkers (had consumed one or more alcoholic drinks in the past 30 days), had purchased alcohol at the liquor store, and did not self-report being pregnant or breastfeeding.

### Measures

#### Noticing labels.

To assess “noticing” the alcohol labels, participants were asked whether they had seen any warning labels on bottles or cans of beer, wine, hard liquor, coolers, or ciders. Responses were dichotomized as “yes” and “no/ don’t know.” The measure at Wave 1 was anchored with 6 months prior, the measure at Wave 2 from November prior to follow-up, and Wave 3 from April prior to follow-up.

#### Recall.

Among those that indicated “noticing,” participants were first asked an unprompted open-ended question to indicate what messages they had seen on the warning labels. Subsequently, to assess prompted recall, participants were shown a list of possible label messages and were asked to select all messages that they saw on alcohol containers. Response options included alcohol and cancer, low-risk drinking guidelines, number of standard drinks in bottles or cans, alcohol may be an addictive drug, alcohol and liver disease, alcohol and trauma, alcohol and fetal alcohol spectrum disorder, and drinking alcohol and driving a car or operating machinery. Both recall measures were anchored similarly to the “noticing labels” measure. For the unprompted recall measure, an RA blinded to experimental conditions coded each response. A second coder reviewed ambiguous responses and discussed them with the first coder to reach consensus. Any reference to drinking guidelines or mention of cancer was coded as recall of national drinking guidelines and recall of the cancer label, respectively.

#### Awareness of national drinking guidelines.

Awareness of the national drinking guidelines was measured by the question, “Were you aware of Canada’s Low-Risk Drinking Guidelines before today?” Responses were dichotomized as “yes” and “no/don’t know.”

#### Knowledge of sex-specific recommended drink limits.

Knowledge of sex-specific daily recommended drink limits was measured by the question, “What is the daily limit of ‘standard drinks’ recommended for males/females (depending on identified sex) in Canada’s Low-Risk Drinking Guidelines?” Participants were asked to enter the number of standard drinks per day. Consistent with the language used in Canada’s Low-Risk Drinking Guidelines (Butt et al., 2011) and on the intervention labels, males who reported up to and including three standard drinks and females who reported up to and including two standard drinks were defined as “correct.” Similarly, knowledge of sex-specific weekly recommended drink limit was measured by the question, “What is the weekly limit of ‘standard drinks’ recommended for males/ females (depending on identified sex) in Canada’s Low-Risk Drinking Guidelines?” Participants were asked to enter the number of standard drinks per week. Males who reported up to and including 15 standard drinks and females who reported up to and including 10 standard drinks were defined as “correct.” The responses were dichotomized as “correct” and “incorrect/don’t know” for knowledge of drink limits.

#### Support for labels with national drinking guidelines.

Participants were asked if cans and bottles of alcoholic beverages should be labeled with Canada’s low-risk drinking guidelines (LRDG). Responses were given on a 5-point Likert scale from 1 = *strongly disagree* to 5 = *strongly agree,* with *don’t know* and *prefer not to say* as options.

#### Sociodemographics.

Sociodemographic measures included age, sex, ethnicity (White, Aboriginal, and other/don’t know/prefer not to say/missing), education (low [completed high school or less], medium [completed trades or college certificate, some university or university certificate below Bachelor’s], high [university degree or post-graduation], and unknown [don’t know/prefer not to say/missing]), and income (low [<$30,000], medium [$30,000–$59,999], high [≥$60,000], and unknown [don’t know/prefer not to say/ missing]). (Income is in Canadian dollars.)

#### Other covariates.

Health literacy was assessed using the Newest Vital Sign assessment tool, a short validated measure to identify health literacy levels (Weiss et al., 2005), and responses were categorized as follows: limited (≤1 correct responses), possibility of limited (2–3 correct responses), adequate literacy (4–6 correct responses), and unknown (don’t know/prefer not to say/missing). Alcohol use was measured using the quantity/frequency method (Heeb & Gmel, 2005). Participants were asked to indicate how often they drank alcoholic beverages in the past 6 months, and how many drinks they usually drank per occasion. Responses were combined to provide a mean number of drinks per week and were categorized using Canada’s LRDG: low (≤10 for females/15 for males per week), risky (11–19/16–29 per week), high (≥20/30 per week) (Butt et al., 2001), and unknown (don’t know/prefer not to say/missing). Last, a time-in-sample variable was created to adjust for participants who participated in one, two, or all three survey waves.

### Statistical analysis

Generalized estimating equation (GEE) models using a binomial distribution with logit link function were used to examine the impact of the intervention labels on five outcomes: unprompted and prompted recall of the drinking guidelines label message, awareness of the national drinking guidelines, and knowledge of daily and weekly drink limits. GEE models can account for a mix of within-subject correlation that arises from the cohort participants being asked the same questions over multiple survey waves, in addition to accounting for the replenishment sample (Pepe, 2003). Difference-in-difference (DID) terms were added to each model to assess the change in outcomes across waves and between sites. The DID terms included an interaction between survey wave and site. Sociodemographics and other covariates were included in all models, with ethnicity defined as White vs. other (Aboriginal/other/don’t know/prefer not to say/missing). Education, income, and health literacy were found to be correlated; thus, to improve the stability of the models, only education was used. The GEE model estimating unprompted recall of the national drinking guidelines label required the addition of a dummy observation to address non-convergence due to a cell count of 0. “Prefer not to say/ missing” responses were excluded from the outcome measures in all models. All analyses were conducted using SAS Version 9.3 (SAS Institute Inc., Cary, NC).

## Results

The final sample consisted of 2,049 unique participants, providing 3,277 observations. Response rates in the intervention and comparison sites were 8.9% and 8.0%, respectively (American Association for Public Opinion Research, 2011), with 53.2% of participants retained at Wave 2 and 47.5% retained at Wave 3. Participants lost to follow-up between waves were more likely to be younger; be male; have lower education, income, and literacy; consume risky, high, or unknown levels of alcohol; and be in the comparison site. Table 1 presents the sample characteristics of participants by site at time of recruitment. The percentage of participants noticing labels was high in all three waves in both the intervention (Wave 1 = 80.4%; Wave 2 = 76.7%; Wave 3 = 80.5%) and comparison (Wave 1 = 87.0%; Wave 2 = 78.5%; Wave 3 = 72.9%) sites.

**Table 1.**
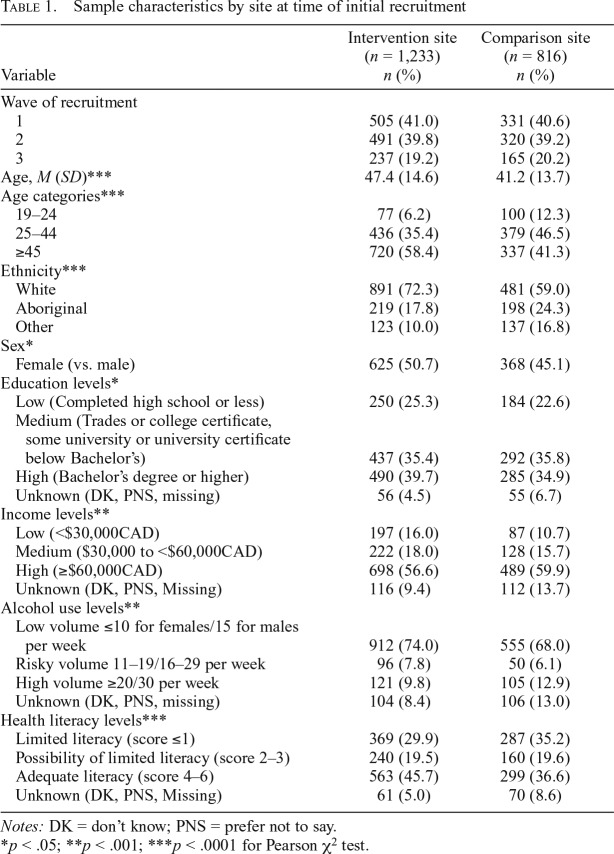
Sample characteristics by site at time of initial recruitment

Variable	Intervention site (*n* = 1,233) *n* (%)	Comparison site (*n* = 816) *n* (%)
Wave of recruitment		
1	505 (41.0)	331 (40.6)
2	491 (39.8)	320 (39.2)
3	237 (19.2)	165 (20.2)
Age, *M* (*SD*)***	47.4 (14.6)	41.2 (13.7)
Age categories***		
19–24	77 (6.2)	100 (12.3)
25–44	436 (35.4)	379 (46.5)
≥45	720 (58.4)	337 (41.3)
Ethnicity***		
White	891 (72.3)	481 (59.0)
Aboriginal	219 (17.8)	198 (24.3)
Other	123 (10.0)	137 (16.8)
Sex*		
Female (vs. male)	625 (50.7)	368 (45.1)
Education levels*		
Low (Completed high school or less)	250 (25.3)	184 (22.6)
Medium (Trades or college certificate, some university or university certificate below Bachelor’s)	437 (35.4)	292 (35.8)
High (Bachelor’s degree or higher)	490 (39.7)	285 (34.9)
Unknown (DK, PNS, missing)	56 (4.5)	55 (6.7)
Income levels**		
Low (<$30,000CAD)	197 (16.0)	87 (10.7)
Medium ($30,000 to <$60,000CAD)	222 (18.0)	128 (15.7)
High (≥$60,000CAD)	698 (56.6)	489 (59.9)
Unknown (DK, PNS, Missing)	116 (9.4)	112 (13.7)
Alcohol use levels**		
Low volume ≤10 for females/15 for males per week	912 (74.0)	555 (68.0)
Risky volume 11–19/16–29 per week	96 (7.8)	50 (6.1)
High volume ≥20/30 per week	121 (9.8)	105 (12.9)
Unknown (DK, PNS, missing)	104 (8.4)	106 (13.0)
Health literacy levels***		
Limited literacy (score ≤1)	369 (29.9)	287 (35.2)
Possibility of limited literacy (score 2–3)	240 (19.5)	160 (19.6)
Adequate literacy (score 4–6)	563 (45.7)	299 (36.6)
Unknown (DK, PNS, Missing)	61 (5.0)	70 (8.6)

*Notes:* DK = don’t know; PNS = prefer not to say.

* *p* < .05;

** *p* < .001;

*** *p* < .0001 for Pearson χ^2^ test.

**Figure 3 (a–e).**
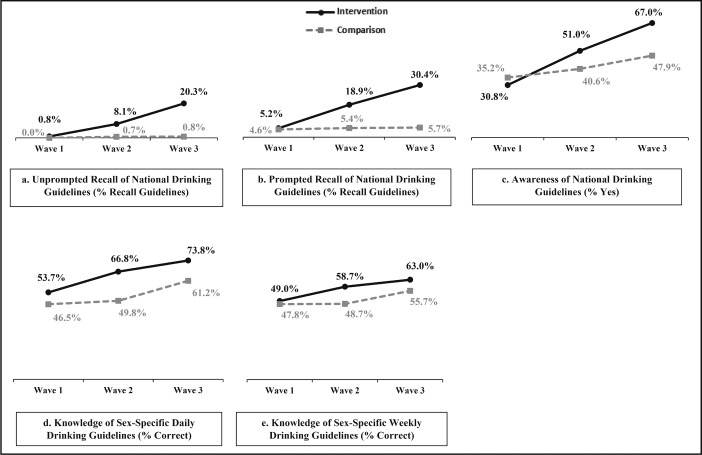
Impact of intervention alcohol labels on outcomes in intervention and comparison sites (Waves 1 to 3), unadjusted %

Unprompted recall of the drinking guidelines label message increased 3.1 times more between Waves 1 and 2 (+7.3% vs. +0.7%, adjusted odds ratio [AOR] = 3.1, 95% CI [0.3, 32.7]) and 10.8 times more between Waves 1 and 3 (+19.5% vs. +0.8%, AOR = 10.8, 95% CI [0.9, 127.6]; Figure 3a, Table 2) in the intervention versus the comparison site. Results of additional GEE modeling comparisons can be found in Supplemental Table A. (Supplemental material appears as an online-only addendum to this article on the journal’s website.)

**Table 2.**
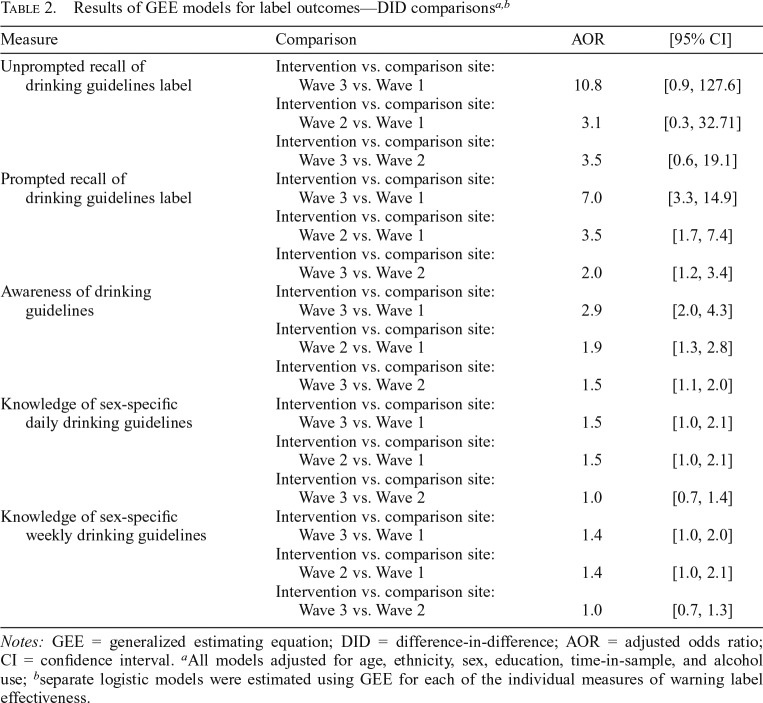
Results of GEE models for label outcomes—DID comparisons*^a^*^*,*^*^b^*

Measure	Comparison	AOR	[95% CI]
Unprompted recall of drinking guidelines label	Intervention vs. comparison site:		
	Wave 3 vs. Wave 1	10.8	[0.9, 127.6]
	Intervention vs. comparison site:		
	Wave 2 vs. Wave 1	3.1	[0.3, 32.71]
	Intervention vs. comparison site:		
	Wave 3 vs. Wave 2	3.5	[0.6, 19.1]
Prompted recall of drinking guidelines label	Intervention vs. comparison site:		
	Wave 3 vs. Wave 1	7.0	[3.3, 14.9]
	Intervention vs. comparison site:		
	Wave 2 vs. Wave 1	3.5	[1.7, 7.4]
	Intervention vs. comparison site:		
	Wave 3 vs. Wave 2	2.0	[1.2, 3.4]
Awareness of drinking guidelines	Intervention vs. comparison site:		
	Wave 3 vs. Wave 1	2.9	[2.0, 4.3]
	Intervention vs. comparison site:		
	Wave 2 vs. Wave 1	1.9	[1.3, 2.8]
	Intervention vs. comparison site:		
	Wave 3 vs. Wave 2	1.5	[1.1, 2.0]
Knowledge of sex-specific daily drinking guidelines	Intervention vs. comparison site:		
	Wave 3 vs. Wave 1	1.5	[1.0, 2.1]
	Intervention vs. comparison site:1		
	Wave 2 vs. Wave 1	1.5	[1.0, 2.1]
	Intervention vs. comparison site:		
	Wave 3 vs. Wave 2	1.0	[0.7, 1.4]
Knowledge of sex-specific weekly drinking guidelines	Intervention vs. comparison site:		
	Wave 3 vs. Wave 1	1.4	[1.0, 2.0]
	Intervention vs. comparison site:		
	Wave 2 vs. Wave 1	1.4	[1.0, 2.1]
	Intervention vs. comparison site:		
	Wave 3 vs. Wave 2	1.0	[0.7, 1.3]

*Notes:* GEE = generalized estimating equation; DID = difference-in-difference; AOR = adjusted odds ratio; CI = confidence interval.

^a^ All models adjusted for age, ethnicity, sex, education, time-in-sample, and alcohol use;

^b^ separate logistic models were estimated using GEE for each of the individual measures of warning label effectiveness.

Prompted recall of the national drinking guidelines label message increased 3.5 times more between Waves 1 and 2 (+13.7% vs. +0.8%, AOR = 3.5, 95% CI [1.7, 7.4]), and 7.0 times more between Waves 1 and 3 (+25.2% vs. +1.1%, AOR = 7.0, 95% CI [3.3, 14.9]; Figure 3b, Table 2) in the intervention site versus the comparison site.

Awareness of the national drinking guidelines in Wave 1, before the alcohol labeling intervention, was 30.8% in the intervention and 35.2% in the comparison site (Figure 3c). The increase in awareness of the national drinking guidelines was 1.9 times greater between Waves 1 and 2 (+20.2% vs. +5.4%, AOR = 1.9, 95% CI [1.3, 2.8]) and 2.9 times greater between Waves 1 and 3 (+36.2% vs. +12.7%, AOR = 2.9, 95% CI [2.0, 4.3]; Figure 3c, Table 2) in the intervention versus the comparison site.

Although knowledge of recommended sex-specific drink limits increased in both sites over the study period, results of the DID analyses showed a 1.5 times greater increase in knowledge of the daily drink limits between Waves 1 and 2 (+13.1% vs. +3.3%, AOR = 1.5, 95% CI [1.0, 2.1]) and 1.5 times greater increase between Waves 1 and 3 (+20.1% vs. +14.7%, AOR = 1.5, 95% CI [1.0, 2.1]; Figure 3d, Table 2) in the intervention versus the comparison site. Similarly, DID results revealed a 1.4 times greater increase in knowledge of the weekly drink limits between Waves 1 and 2 (+9.7% vs. +0.9%, AOR = 1.4, 95% CI [1.0, 2.1]) and a 1.4 times greater increase between Waves 1 and 3 (+14.0% vs. +7.9%, AOR = 1.4, 95% CI [1.0, 2.0]; Figure 3e, Table 2) in the intervention versus the comparison site.

To test the contribution of including a label with a cancer warning alongside a label with the national drinking guidelines, GEE models estimating the relationships between recall of the cancer message, either unprompted or prompted, and awareness and knowledge of drinking guidelines were conducted, adjusting for sociodemographics and other covariates. The results indicated that those who recalled the cancer message were 2.0 times more likely to be aware of the drinking guidelines (AOR = 2.0, 95% CI [1.6, 2.4]), and 1.6 and 1.3 times more likely to know the daily (AOR = 1.6, 95% CI [1.3, 1.9]) and weekly (AOR = 1.3, 95% CI [1.0, 1.5]) drink limits, respectively, compared with those who did not recall the cancer message.

**Figure 4.**
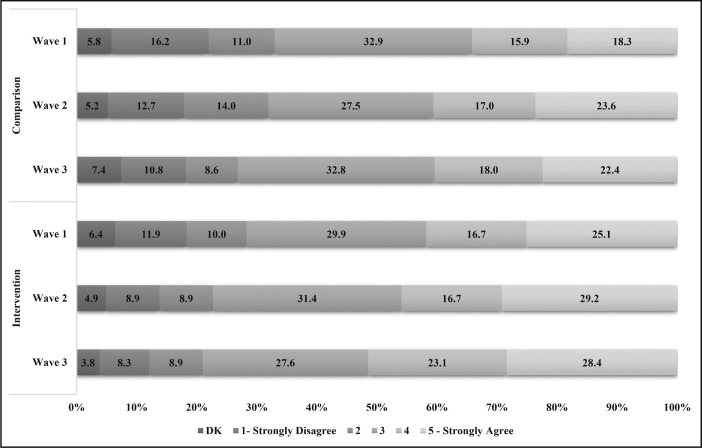
Degree of support for national drinking guidelines labels on alcohol containers, unadjusted %. DK = don’t know.

The majority of participants were neutral to strongly supportive of applying labels with national drinking guidelines on alcohol containers in both the intervention (Wave 1 = 71.7%; Wave 2 = 77.3%; Wave 3 = 79.1%) and comparison (Wave 1 = 67.1%; Wave 2 = 68.1%; Wave 3 = 73.2%) sites (Figure 4). See Supplemental Figure A for a visual summary of results.

## Discussion

Alcohol drinking guidelines are used internationally to provide evidence-informed recommendations of upper drink limits for low-risk alcohol consumption. To adhere to these drinking guidelines, consumers must be aware of and understand the recommended limits. International research indicates that public awareness and knowledge of drinking guidelines is lower than 50% in most jurisdictions (Bowden et al., 2014; Buykx et al., 2018; De Visser & Birch, 2012; Livingston, 2012; Rosenberg et al., 2018). Experts suggest that providing directional information on how to use alcohol is crucial for the consumer and should accompany the sale of all alcohol products as a public health promotion message (Cancer Council Australia, 2013). Results of the current study demonstrate that enhanced alcohol labels may be an effective tool for increasing awareness and knowledge of drinking guidelines. Before the labeling intervention, one in three consumers in the current study were aware of Canada’s LRDG. Following the intervention, awareness more than doubled in the intervention site, an increase that was more than two times greater among those exposed versus those unexposed to the alcohol labels. This increase in awareness over the study period is consistent with the proportion of participants that recalled the drinking guidelines on the label, either unprompted or prompted, confirming the label’s role in boosting participants’ awareness of national drinking guidelines in the current study.

Modest increases in knowledge of the daily and weekly drink limits were also observed following exposure to enhanced alcohol labels in the intervention site, both in the shorter term (Waves 1 to 2) and over the full study period (Waves 1 to 3). One explanation for the modest differences in changes in knowledge between sites is that knowledge of the daily and weekly guidelines increased in both the intervention and comparison sites over the study period. Increases among participants in the comparison site not exposed to the label intervention are likely because the study received national and international media coverage following the alcohol industry’s interference in the study (Austen, 2018; Joannou, 2018; Vallance et al., 2020a; Vallance et al., 2020b). Although the media largely focused on the cancer warning label, images of the labels with the cancer warning and national drinking guidelines were circulated in the media, and it is possible that the media elicited extra attention to alcohol-related health harms and safer drinking recommendations, including Canada’s LRDG. It is worth noting, however, that the intended social marketing campaign was not implemented in the intervention site during the intervention period due to the industry’s interference, and the media coverage may have partly replaced the function of the canceled campaign in the intervention site.

Nevertheless, the results of the current study demonstrate the unique benefits of labels for increasing awareness and knowledge of national drinking guidelines. In addition, the study provides evidence that recalling the cancer label message enhanced the intervention impact on LRDG outcomes, highlighting the potential synergistic effects of the rotating labels. Previous population-based interventions that were intended to increase awareness and knowledge of drinking guidelines focused on longer multicomponent information-based campaigns, with various levels of success. Two of these campaigns, one conducted in the Canadian province of Quebec and the other in Denmark, reported increases in knowledge of drink limits following much longer-term and well-resourced campaigns (Éduc’alcool, 2016, 2019; Grønbaek et al, 2001). However, these interventions lacked a comparison group, making it difficult to control for secular influences. Further research is needed to test the impact of an uninterrupted longer-term labeling intervention on awareness and knowledge of drinking guidelines, as well as the impact on actual drinking behavior.

Previous studies have highlighted the association between knowledge of drinking guidelines and lower levels of alcohol use (Bowring et al., 2012; Coomber et al., 2017; Islam et al., 2019), but the underlying mechanisms for this association are not well understood. It is well documented that labels are likely a key component of a comprehensive alcohol strategy to increase consumer awareness of alcohol-related risks and knowledge of safer alcohol use, and ultimately reduce alcohol consumption and harms (Centre for Addiction and Mental Health, 2019; Martin-Moreno et al., 2013; WHO, 2017). Last, the results of the current study show that labels with national drinking guidelines are unlikely to be received negatively among drinkers, as participant support was high in Wave 1 and remained high across the study period in both sites. This is in line with previous research indicating support for drinking guidelines on alcohol labels (Coomber et al., 2017).

The study has several limitations. First, considering that the intervention was interrupted and shortened due to the alcohol industry’s interference and the small sample sizes in both sites, the effects of the labels might have been stronger if these limitations had been resolved and the study had been implemented as planned. Next, the study sample was not representative of the site populations, as participants were recruited from liquor stores in city centers using non-probability-based methods, limiting generalizability. In addition, the national media coverage of the alcohol industry’s interference in the study may have contaminated the comparison site by exposing information about the study and intervention, particularly the alcohol label messages. Finally, due to very low rates of unprompted recall in the comparison group, DID results lacked precision and produced wide CIs. However, the low rates of recall and awareness of the national drinking guidelines before the alcohol label intervention also provide a strong rationale for enhanced alcohol labels.

### Conclusions

Alcohol labels with national drinking guidelines, standard drink information, and a cancer warning may be an effective population-level strategy for increasing awareness of drinking guidelines and knowledge of drink limits, a stated goal of international alcohol control efforts (Australia Department of Health, 2019; CCSA, 2007; UK Department of Health, 2007). This study also supports previous claims that labels with drinking guidelines have high levels of public support. Alcohol labels should aim to maximize population reach and expand on the single pregnancy messages currently used in many jurisdictions.

## Acknowledgments

The authors would like to acknowledge all the RAs who helped collect the data, as well as the liquor control boards, health and social services, and community partners in Yukon and Northwest Territories for their commitment and support in developing and executing this research. Special thanks also go to Mark Petticrew and Melanie Wakefield for their expertise and guidance.
